# Deep learning based lithology classification of drill core images

**DOI:** 10.1371/journal.pone.0270826

**Published:** 2022-07-01

**Authors:** Dong Fu, Chao Su, Wenjun Wang, Rongyao Yuan

**Affiliations:** College of Water Conservancy and Hydropower Engineering, Hohai University, Nanjing, China; Universiti Malaysia Pahang, MALAYSIA

## Abstract

Drill core lithology is an important indicator reflecting the geological conditions of the drilling area. Traditional lithology identification usually relies on manual visual inspection, which is time-consuming and professionally demanding. In recent years, the rapid development of convolutional neural networks has provided an innovative way for the automatic prediction of drill core images. In this work, a core dataset containing a total of 10 common lithology categories in underground engineering was constructed. ResNeSt-50 we adopted uses a strategy of combining channel-wise attention and multi-path network to achieve cross-channel feature correlations, which significantly improves the model accuracy without high model complexity. Transfer learning was used to initialize the model parameters, to extract the feature of core images more efficiently. The model achieved superior performance on testing images compared with other discussed CNN models, the average value of its Precision, Recall, *F*_1−score_ for each category of lithology is 99.62%, 99.62%, and 99.59%, respectively, and the prediction accuracy is 99.60%. The test results show that the proposed method is optimal and effective for automatic lithology classification of borehole cores.

## 1 Introduction

In the field of underground engineering, knowing the geological conditions around the project mainly relies on geological drilling. The extracted core provides the most reliable lithology information [1], and the lithology of the drill core reflects the lithology of the geological structure in the drilling area. The lithological data determines the location of the underground project and the orientation of the axis of the underground structure in the early stage of construction and provides a valuable reference for the treatment of adverse geological conditions during the construction process. Core analysis is usually manual visual inspected by geologists, who will identify the lithology according to the color, structure, and texture of the rock, as well as mineral composition [2]. It requires practitioners to have high professional quality and extensive fieldwork experience. The work is not only labor-intensive and time-consuming [3], but also very subjective. In addition to identification with the naked eye, geologists also use some physical testing methods to help detect, such as X-ray, CT scan, electron microscope, and isotope methods [4–6], etc.

In recent years, the rapid development of computer technology as well as data science, especially data storage, and low-cost computing (the popularity of GPU) have made large-scale arithmetic available [7]. Currently, artificial intelligence techniques have been widely used in the field of underground engineering, such as concrete crack detection [8, 9], the detection of various defects on concrete surfaces, the identification of rock mass structures in tunnels and underground structures excavation [10], rock fragment classification [11], structural health monitoring [12], and tunnel surface settlement prediction [13], etc. These methods provide new solutions to a series of difficult problems in relatively traditional engineering fields.

Due to the complex structure and texture in rocks, and the mineral composition of rocks is diverse, resulting in a wide variety of rocks and making lithology identification difficult. The use of well log data to identify lithology is currently the mainstream method. Conventional log data include: sonic, neutron, gamma, density, additionally, the prompt gamma neutron activation analysis (PGNAA) can also assist lithology interpretation, and enhance lithology prediction [14]. The tools used for lithology identification are mainly various machine learning algorithms [15]. Shan et al. [16] selected two logging curves, natural gamma and photoelectric absorption cross-section index, which are more sensitive to lithology, and used BP neural network to identify complex lithology with good results. Yang et al. [17] used 12 kinds of physical logging parameters such as volcanic rock fabric and pore structure as the classification feature quantities, and the AdaBoost-decision tree algorithm was used to classify volcanic lithology with 90% accuracy. Al-Mudhafar [18] used the generalized boosted regression model (GBM) to establish a nonlinear relationship among well logging data, lithofacies, and core permeability to obtain lithofacies classification. The results showed that the GBM algorithm significantly outperformed the traditional multiple linear regression. Antariksa et al. [19] used two classifiers, random forest and gradient boosting, for petrographic classification of log data from the Tarakan basin, and achieved more reliable results compared to other machine learning methods. In addition, support vector machines (SVM) have also been proven to have great potential in rock lithology identification [20, 21].

Traditional well log data are one-dimensional and contain limited rock information. It is a better choice to use image logs for lithology identification because they are two-dimensional data, which naturally provide more rock information. One of the methods took the RGB or gray-scale image of the rock slice as input and obtained multiple numerical feature values through image processing, which was used as input to train the multilayer perceptron [22] network to achieve rapid recognition of rock textures [23, 24]. Chai et al. [25] used a watershed algorithm to extract features from the input borehole images and perform feature selection and finally used it to discriminate the lithology. Although the results achieved by these methods are considerable, the rock features are still processed manually [26], the efficiency of feature extraction is not high and the reliability of the extracted features is not objective. Lithology recognition lacks intelligence, and the recognition accuracy needs to be improved.

Based on these issues, researchers have shifted their attention to deep neural networks (Deep learning) [27]. With the growth of computing power, deep learning methods, especially convolutional neural networks (CNN) [28], have been "rediscovered" in the last decade and have become absolutely dominant in the field of computer vision. A series of open-source CNN architectures built by statistical modelers, such as AlexNet [29], VGG [30], GoogLeNet [31], and DenseNet [32], etc., enable researchers in different fields to use a wide variety of networks to solve problems in their respective fields. Compared to manual feature extraction, the convolutional kernel of CNN can automatically extract deep-level features [33]. Thomas et al. [1] used gray-scale images of drill cores to classify three lithologies, carbonate-cement, shale and sandstone, and obtained a classification accuracy of 94%, which is an early attempt to predict lithology from drill core images. Zhang et al. [34] identified three selected lithologies, sandstone, shale, and conglomerate, using convolutional neural networks on a dataset of 1500 on a two-dimensional gray-scale image dataset of 64 pixels in height and width and obtained 95% prediction accuracy. Valentín et al. [35] used ultrasonic and microresistivity borehole image logs as inputs, and utilized a deep residual convolutional network to extract features and then infer the lithology of each sample. Obtained an average classification accuracy of 81.45% on the blind test sample. It could be concluded that most of their work focus on relatively simple gray-scale images, or images generated from well log data, and not enough on the RGB images of the borehole cores themselves.

Baraboshkin et al. [36] collected 2000 m cores from different regions of Russia and preprocessed them to obtain lithology in six categories: massive and laminated sandstone, limestone, granite, shale, and siltstone, with a total of 20,000 drill core images of 10×10 cm, several well-known CNN architectures were used, among which better results were achieved on GoogLeNet [31], achieving 72% accuracy on new core images, which is two percentage points higher than ResNet [37]. In a recent study by Alzubaidi et al. [38], they obtained more than 800 core tray images from a total of 28 boreholes in Australia, and cropped them into 76,500 RGB images, containing sandstone, limestone, and shale three kinds of lithology, in addition, the fourth ‘garbage’ class was added to characterize the non-core portion of the borehole core. The more advanced ResNeXt-50 model [39] was tested on these input images, which outperformed the ResNet-18 [37] and Inception-v3 [40] in terms of performance, achieving a prediction accuracy of 93.12% on new core images. Besides, the use of CNN to extract thin-section image features of rocks to classify rocks [41, 42] has also been proven to have very good results, providing a new thought for rock classification. Deep learning requires a large number of training samples as support [43]. Although their work collected a large number of drill core images from different regions of their respective countries, the number of lithology categories involved in the dataset is relatively small, with the number of categories less than six, and these data are not publicly available. Compared with rocks with complex structures and many lithology types involved, it is difficult to apply the trained network to other borehole images, and its applicability to different drill core images is greatly limited.

**Fig 1:**
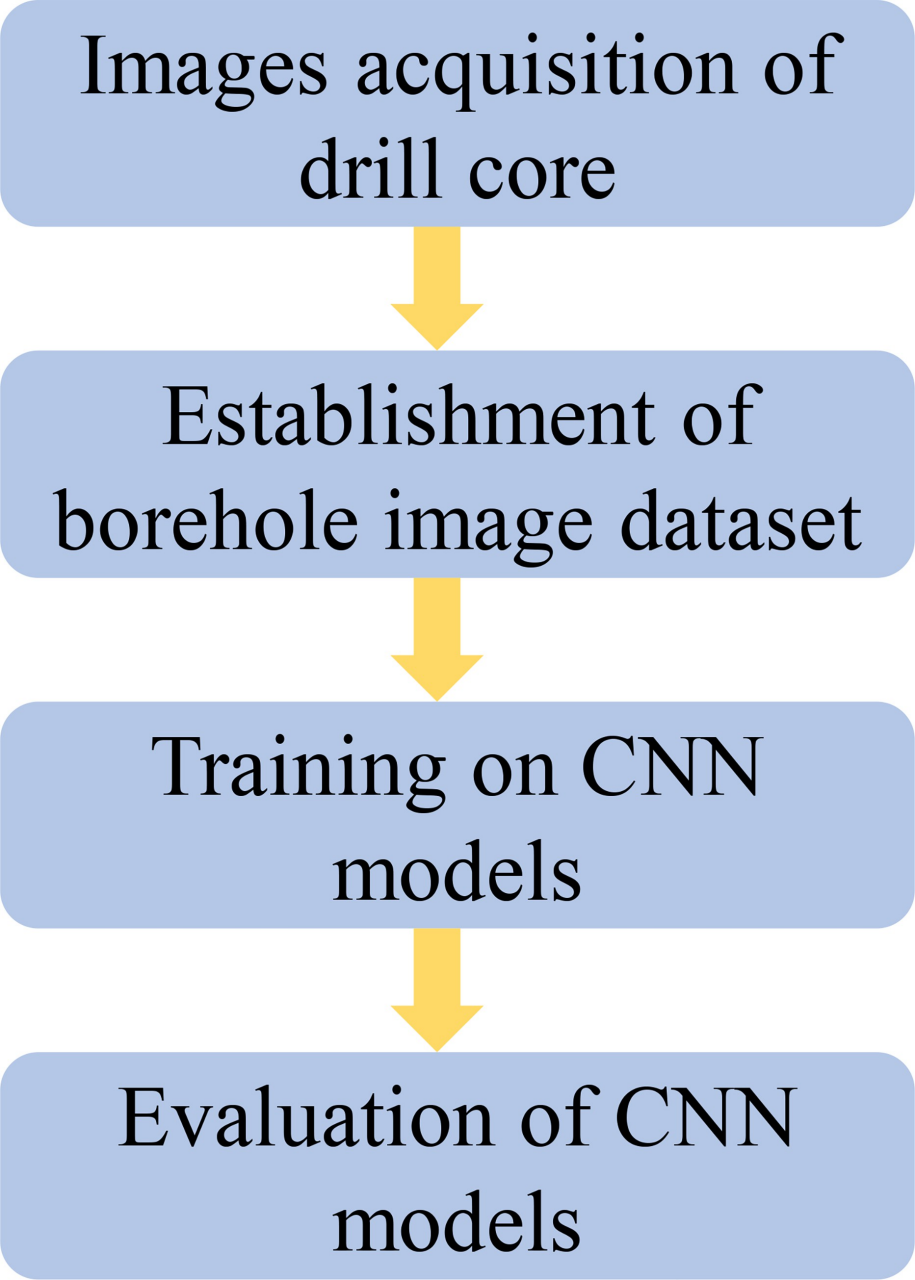
Flowchart of the overall procedure of the proposed method.

In this work, we collected many borehole images and established a dataset of drill core lithology images in the field of underground engineering. The dataset includes most of the common lithologies in underground engineering, involving more types of lithology with a total of 10 categories, which is higher than the previous existing dataset, and the trained classifier is more abundant in the application scenarios. The more advanced ResNeSt-50 [44] was used and details of the learning are presented to intelligently classify these core images. To compare model performance, the results are compared with the ResNet-50, DenseNet-161, and VGG-13 architectures. Fig 1 summarizes the workflow of lithology classification of drill core images. Section 2 presents the core images acquisition as well as the data processing details to build the image dataset. Section 3 describes the different CNN architectures, and the details of training, furthermore, the evaluation metrics for assessing the framework are also introduced. Section 4 evaluates the performance of different CNN models from different perspectives, and finally uses Grad-CAM [45] to visualize CNN. Sections 5 and 6 are the discussion and conclusion parts, respectively.

## 2 The dataset

Geological drill cores are the most direct reflection of geology. There are relatively few open-source datasets related to borehole cores in underground engineering. One reason is that drilling the strata is very expensive, the drilling cycle is relatively long, and not all drilling can obtain satisfactory cores. Furthermore, the work of strata drilling is often done by independent geological departments, and it is very difficult for others to get these data because of the interests involved.

### 2.1 Images acquisition

In this work, all data are sourced from the China Geological Sample Information (CGSI), where the data sources are reliable and can be obtained publicly. By visiting the CGSI website, the borehole image data can be downloaded by selecting the appropriate core borehole. The resolution of the downloaded original borehole images is not fixed, and the image is in JPG format. In this work, 10 rock categories that are more common in underground engineering hydraulic facilities were collected, namely diabase, diorite, gneiss, medium-coarse-grain biotite granite (hereinafter referred to as granite), limestone, marble, monzonite, mudstone, shale, and siltstone. They were obtained from hydraulic fracturing in-situ stress measurement boreholes, hydrogeological exploration boreholes, and a few other geological boreholes, totaling 18 boreholes, each with a depth of a few hundred meters to over a thousand meters.

In these boreholes, each core image contains only a longitudinally placed core with different lengths, and a complete core consists of hundreds or even thousands of such images. The lithology categories to which the images belong were grouped according to the reliable lithology logs given by experienced geologists. Since these pictures were taken uniformly, there were many blurred images among them, which were discarded in this article. In this way, a complete drill core must have many discontinuities. These boreholes are usually drilled by different geological departments, so the diameters of these cores are not consistent, and there is no evidence for the diameters of the drill cores in CGSI, but all cores’ diameters are certainly larger than 3 cm. The different diameters of the cores result in images of varying sizes and resolutions, making it difficult to crop the images to the exact length.

### 2.2 Crop size

In this paper, core images were cropped based on the pixel size for the convenience of cropping. Appropriate input image size is very important for the CNN model. Small-scale images contain fewer lithology features, while large-scale images may contain many separated fractures. In the work of Alzubaidi [38], they tested the sizes of 60 × 60 pixels, 120 × 120 pixels, 180 × 180 pixels, and 240 × 240 pixels to crop each core image, and the pre-trained ResNet-18 was retrained. The test results showed that larger crop sizes are generally better, include more lithology information, and have lower losses in training and validation. The scale of the cropped image in this article is 256 × 256 pixels, which corresponds actual scale is around 3 × 3 cm. In the following sections, all models were trained based on 256 × 256 pixels images.

### 2.3 Image cropping

**Fig 2:**
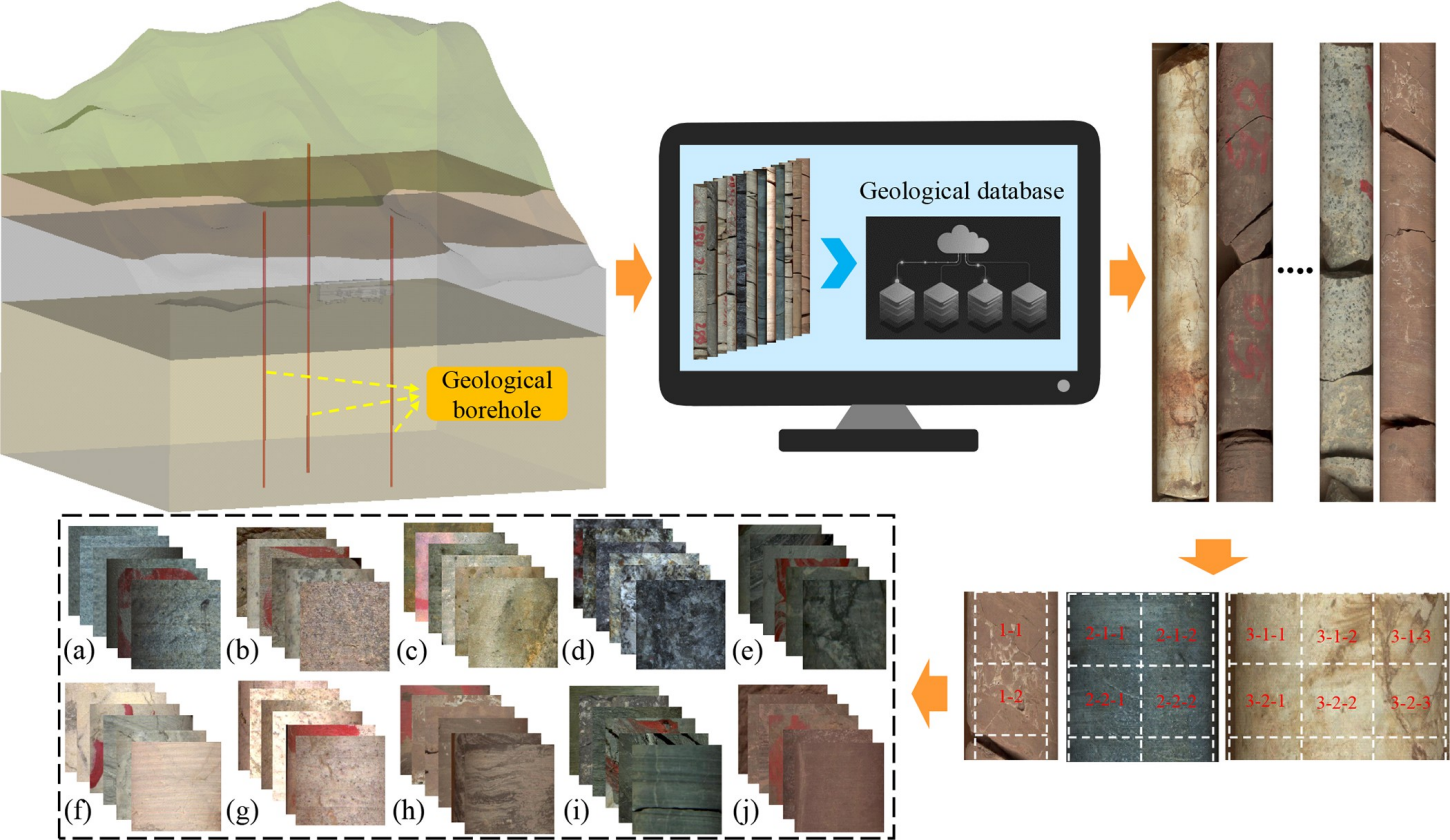
Demonstrate the automated processing of core images. Drill core images and lithology logs obtained from the geological drill holes were first uploaded to the geological database. The core images were gained from the database, and the cropping program made a judgment on the image and cropped it by center cropping. In total, 10 lithology categories were obtained, namely: (a) Diabase; (b) Diorite; (c) Gneiss; (d) Granite; (e) Limestone; (f) Marble; (g) Monzonite; (h) Mudstone; (i) Shale; and (j) Siltstone.

For the core images obtained from CGSI, the core does not fill the whole image, and there are empty tray areas or other irrelevant lithology backgrounds on both sides of some images. This phenomenon exists in every category of rock, which is likely to cause misjudgement of the network. Therefore, it is significant to make the cropped images include more core itself and less background. To this end, we have studied and demonstrated a procedure for automatically processing core images shown in Fig 2. It took the borehole images as input, and made a judgment on the size of these images, using a center cropping strategy and discarding portions that didn’t satisfy the cropping size on both sides, which usually involve useless tray backgrounds.

### 2.4 Dataset preparation

**Fig 3:**
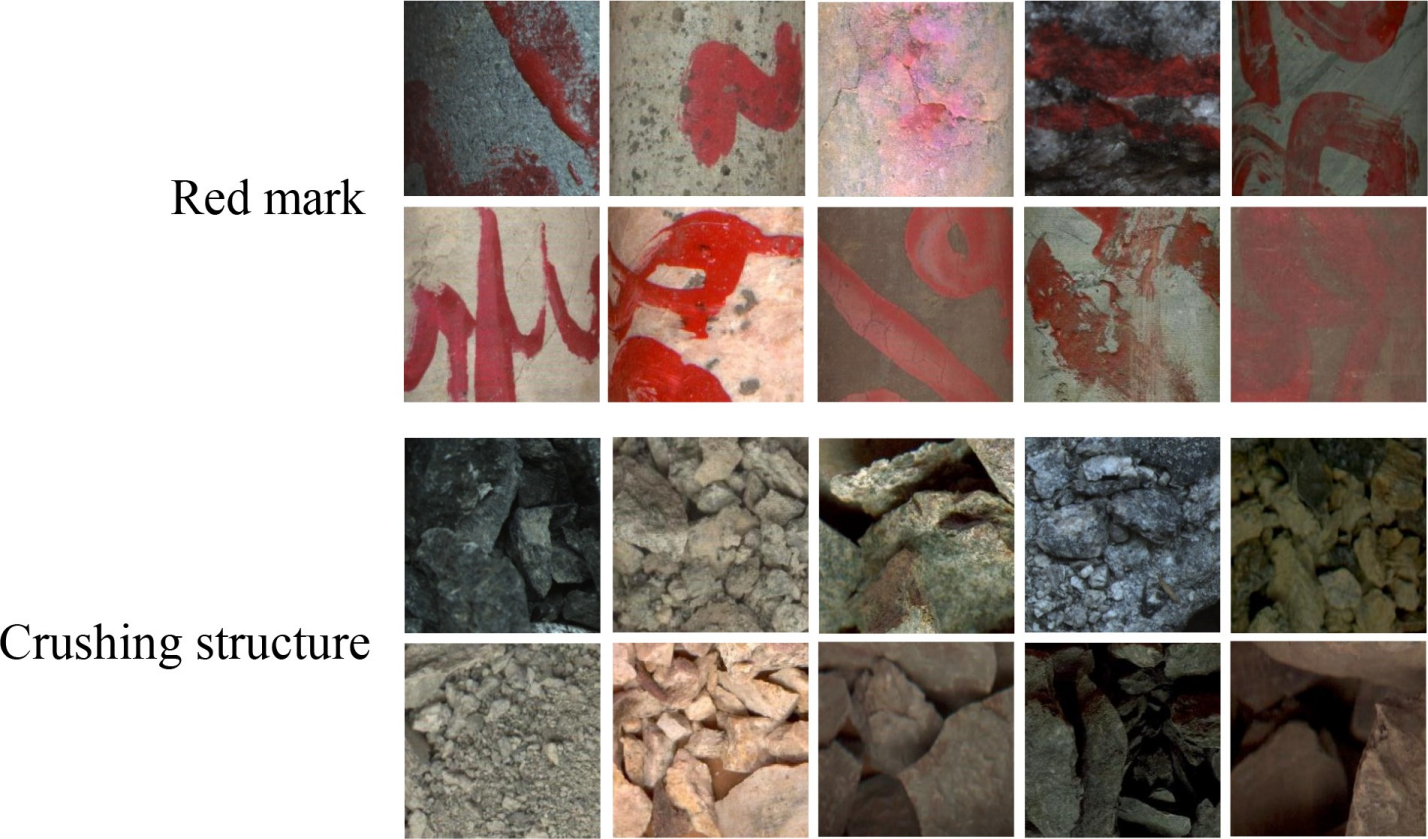
Example of non-core sections images. The parts were easy to cause misclassification by the CNN model, such as red marks and crushing structures.

The images processed by the automatic core processing program contain more core itself. However, there are usually many red marks made by geologists to mark lithology or depth on the core, which is not helpful for model training. In addition, there are also severely fractured rocks in the core section, which form crushed structures. These structures usually contain impurities other than their own lithology, such as sediment particles, tree roots, etc. An example of the image is shown in Fig 3. In addition to the lithology that needs to be classified, it is one way to add an extra ‘garbage’ class to all lithology categories to absorb these non-core parts [38].

**Fig 4:**
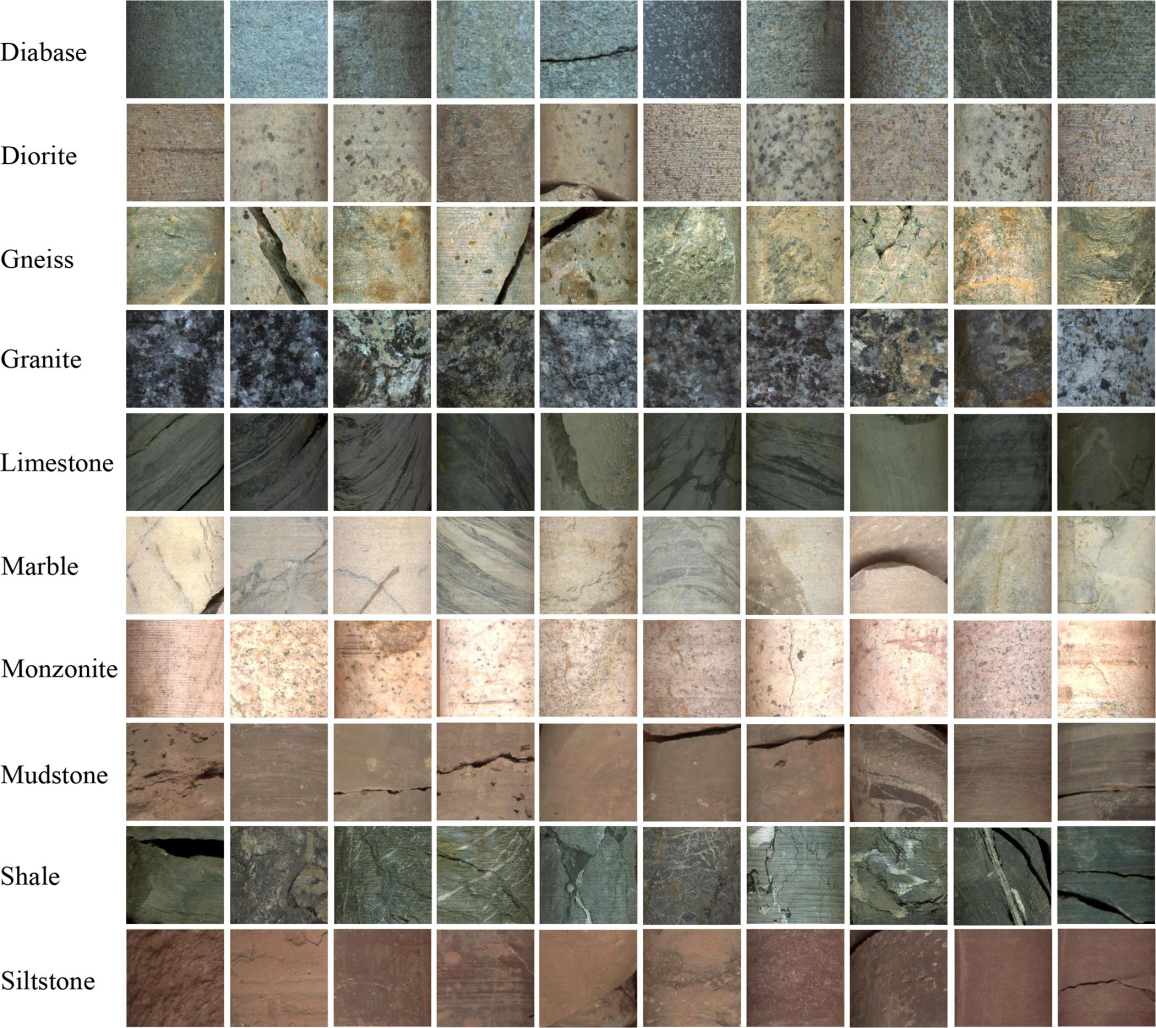
Ten typical samples in each label. The non-core itself was discarded and the core images were annotated according to the geologist’s lithology logs.

In this work, 10 categories of lithology were collected, and the dataset is better at diversity, so these non-core parts were discarded. The filtered images only contain the core itself. Finally, these filtered images were further checked manually with the lithology logs given by geologists, and the bad examples were removed to ensure all images were correctly labeled. A total of 15,000 images were finally obtained, with a total of 1,500 images for each label. Fig 4 demonstrates typical image samples of each category. The images of each label were randomly divided into three parts of training, validation, and testing groups according to the proportion of 7:1:2. Therefore, 1050 and 150 images in each lithology category were used for training and validation, respectively. The training group participates in the training process and is used to update the weight parameters within the model. The validation group doesn’t participate in the training process and is used to check the state of the model during the training process, such as whether the model has converged, whether overfitting has occurred, etc., and is also used to adjust the model hyperparameters. The testing group also doesn’t participate in the training process and should only be used once after the model training is completed to evaluate the generalization performance of the model.

## 3 Methodology

### 3.1 Convolution neural network

Compared with traditional multi-layer perceptron and machine learning algorithms, CNN has more advantages. It can retain the spatial position information of the image, and is insensitive to the noise in the pixel matrix, enabling the extraction of image features in a more efficient way. The CNN architecture consists of alternating combination of convolutional layers, activation layers, and pooling layers. The convolution operation is first performed in convolutional layers, the result of which is nonlinearly operated in activation layers, and finally, the information is aggregated in pooling layers to reduce the sensitivity of the convolutional layer to position and spatial downsampling representation.

#### 3.1.1 The CNN architecture

Because of the extensive use of modular structures (bottleneck) in ResNet [37], its structure is concise and general. Even now, many downstream vision tasks still use ResNet or its variants as the backbone network. For this reason, a similar modular structure that applies channel attention to different network branches for cross-feature interaction was proposed in the latest work of Zhang et al. [44], and the model was named ResNeSt, finally. It outperforms state-of-the-art CNN models generated by neural architecture search [46] in terms of accuracy and latency trade-off for image classification. ResNeSt-50 achieves 81.13% top-1 accuracy on ImageNet [47], which shows the powerful performance of the model.

**Fig 5:**
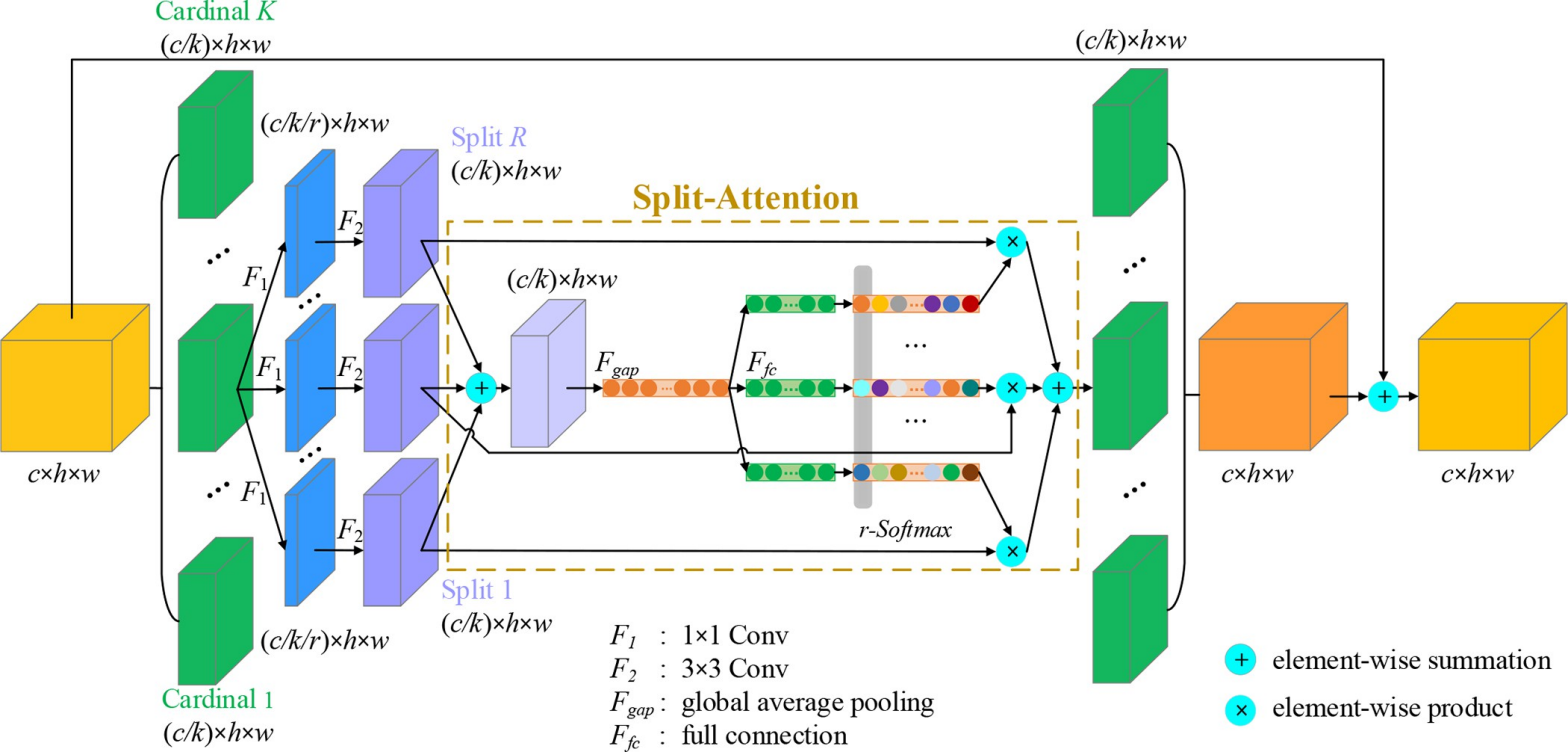
The structure of ResNeSt block. *c*,*h*,*w* are the number of channels, height, and width of the input featuremap, respectively. Cardinal *k* is the *k-*th cardinal group and Split *r* is the *r-*th split.

It is self-evident that multi-path representation and featuremap attention for visual recognition tasks are important. The successful application of multi-path representation on GoogleNet [31] inspires researchers to parallelize the input into multiple paths and extract features with different sized kernels in different paths. ResNeXt [39] enables the multi-path structure to be converted into uniform operation by using group convolutions in the ResNet bottle block. In addition, the channel attention mechanism introduced by SE-Net [48] and the featuremap attention introduced by SK-Net [49] through two network branches have also deeply inspired the authors. Therefore, the final architecture adopted a strategy of combining channel-wise attention with multi-path network. The Split-Attention block (ResNeSt block) in the architecture is shown in Fig 5. In the ResNeSt block, the featuremap is divided into *K* branches (cardinal groups) along the channel dimension, and in each cardinal group, it is divided into *R* subgroups (split), so the total number of feature groups is *G* = *RK*.

The same Split-Attention module is used in each split, as shown in the yellow dashed box in Fig 5. The combined feature ${\hat{U}}^{k}$ of each cardinal group can be obtained by fusing via an element-wise summation across *R* splits.

$${\hat{U}}^{k}=\sum_{m=R(k-1)+1}^{Rk}U_{m}$$ (1)

Where, ${\hat{U}}^{k}\in R^{C/K\times H\times W}$ for *k*∈1,2,⋯,*K*; *m*∈1,2,⋯,*RK*; *C*/*K*, *H*, *W* denote the size of the intermediate featuremap; *U*_*m*_ denotes the *m*-th input feature in the Split-Attention block.

On this basis, the global contextual information of the channel is obtained by global average pooling across the spatial dimensions *s*^*k*^∈ℝ^*C*/*K*^ [49]. The calculation formula of the c-th channel component:

$$s_{c}^{k}=\frac{1}{H\times W}\sum_{x=1}^{H}\sum_{y=1}^{W}{\hat{U}}_{c}^{k}(x,y)$$ (2)

Where, ${\hat{U}}_{c}^{k}(x,y)$ denotes the value of the pixel (*x*, *y*) in the c-th channel of ${\hat{U}}^{k}$.

After *s*^*k*^ is transformed by dense layer, the (soft) assignment weight of a split is calculated by Eq 3.

$$w_{n}^{k}(c)=\left\{ \begin{array}{l} \frac{exp(g_{n}^{c}(s^{k}))}{\sum_{m=1}^{R}exp(g_{m}^{c}(s^{k}))}\;if\;R>1,\\ \frac{1}{1+exp({-g}_{n}^{c}(s^{k}))}\;if\;R=1 \end{array}\right.$$ (3)

Where, $w_{n}^{k}(c)$ is the assignment weight of the c-th channel in the n-th split; $g_{n}^{c}$ is the attention weight function composed of two dense layers and ReLU activation function.

Finally, the weighted fusion feature *V*^*k*^∈ℝ^*C*/*K*×*H*×*W*^ of each cardinal group is generated by weighted combination of each split featuremap and assignment weight. The calculation formula of the c-th channel is following:

$$V_{c}^{k}=\sum_{n=1}^{R}w_{n}^{k}(c)U_{R(k-1)+n}$$ (4)

Where, $V_{c}^{k}$ denotes the weighted fusion feature of the c-th channel in each cardinal group; *U*_*R*(*k*−1)+*n*_ denotes the featuremap of the (*R*(*k*−1)+*n*)-th split. Finally, the cardinal group representations are concatenated along the channel dimension to get: *V* = *Concat*{*V*^1^, *V*^2^,⋯,*V*^*k*^,⋯*V*^*K*^}. Similar to the residual block of ResNet, the final output of the ResNeSt block uses a shortcut connection: *Y* = *V*+*X*. ResNeSt-50, ResNeSt-101, ResNeSt-200, ResNeSt-269 architectures with different depths can be attained by stacking different numbers of ResNeSt blocks.

Due to the complex structure and texture in rocks, and diverse mineral compositions, resulting in a wide majority of rocks and difficult lithology identification, ResNeSt-50 was used as the benchmark network.

### 3.2 Details of training

In this work, the conclusions obtained were derived from computer experiments. Computer hardware equipment includes an Intel(R) Core(TM) i7-8700 CPU @3.20 GHz with 6 cores and 12 logical processors, 4 DDR4 RAM of 8 GB and one NVIDIA GeForce RTX 3060 GPU with 3584 CUDA cores, 12GB GDDR6 memory, and 1867MHz core frequency. The software platform is a 64 bit Windows 10 system and Python 3.8.12 [50] interface based on PyTorch 1.10.2 deep learning framework and OpenCV framework [51].

#### 3.2.1 Loss function

The checked images are fed into the network along with their corresponding labels. After a series of feature extraction operations, the final obtained featuremap information is compressed into one dimension by global average pooling and then linked to the fully connected layer, and the overall probability distribution is output in the softmax layer. The loss function reflects the deviation between the predicted value and the true value and will guide the optimization of parameters during network training to maximize the probability of the predicted result. The loss function with the L2 regularization term is introduced:

$$L(\phi )=P(\phi )+Q(\phi )=-\frac{1}{u}\sum_{i=1}^{u}\sum_{j=1}^{v}\log\frac{exp(o_{j})}{\sum_{w=1}^{v}\exp\left(o_{w}\right)}+\frac{\lambda }{2}{\left\|\phi \right\|}^{2}$$ (5)

Where, *P*(*ϕ*) is the cross-entropy loss with batch size *u*; *Q*(*ϕ*) is an L2 regularization term that is added to the original loss function to penalize large components of the weight vector to reduce overfitting; *ϕ* is the optimizable parameters of the network such as weight, bias; *v* is the number of neurons in the output layer corresponding to the number of label class; *o*_*j*_(1≤*j*≤*v*) is the output value of the j-th neuron in the final dense layer; ‖*ϕ*‖^2^ is the 2-norm of *ϕ*; *λ* is the weight decay coefficient. In this paper, *v* = 10, *λ* = 0.001, and the value of *u* will be discussed in Section 3.2.6.

#### 3.2.2 Optimization algorithm

During the training of the model, the optimization algorithm constantly updates the model parameters to reduce the loss function value, so the performance of the optimization algorithm will directly affect the efficiency of the model training. In this article, AdamW algorithm [52] in Eq 6 was used:

$$\psi_{t+1}=\psi_{t}-\eta \left(\frac{{\hat{m}}_{t}}{\sqrt{{\hat{v}}_{t}}+\epsilon }+\lambda \psi_{t}\right)$$ (6)

Where, *t* is timestep, its initial value is 0; *η* is learning rate; *m*_*t*_, *v*_*t*_ are the first moment and second moment of the deviation estimated by exponentially weighted moving average, respectively. The update methods of *m*_*t*_, *v*_*t*_ are shown in Eqs 7 and 8, and ${\hat{m}}_{t},{\hat{v}}_{t}$ are the deviation correction; *ϵ* is a constant to maintain numerical stability (setting to 1e−8). In short, AdamW is the Adam [53] that introduces an L2 regularization term to limit the parameter values from being too large. *λ* is weight decay coefficient (setting to 1e−3).

$$m_{t}=\beta_{1}m_{t-1}+(1-\beta_{1})∙\nabla_{\psi }f_{t}(\psi_{t-1})$$ (7)

$$v_{t}=\beta_{2}v_{t-1}+(1-\beta_{2})∙\nabla_{\psi }^{2}f_{t}(\psi_{t-1})$$ (8)

Where, ∇_*ψ*_*f*_*t*_(*ψ*_*t*−1_) is gradient; *β*_1_, *β*_2_ are the exponential decay rates of first moment and second moment estimate, respectively, setting to 0.9 and 0.999. *m*_0_, *v*_0_ are initialized to 0. The correction method for the deviation of *m*_*t*_, *v*_*t*_ is shown in Eqs 9 and 10.


$${\hat{m}}_{t}=\frac{m_{t}}{1-\beta_{1}^{t}}$$ (9)

$${\hat{v}}_{t}=\frac{v_{t}}{1-\beta_{2}^{t}}$$ (10)

#### 3.2.3 Learning rate scheduler

During model training, the learning rate determines the degree of model parameters adjustment. If the learning rate is too small, the convergence will be slow, and the training will take a long time. On the contrary, if the learning rate is too large, the loss will oscillate or even become larger, and ultimately the superior result will not be achieved. In this paper, the learning rate strategy used is Cosine Annealing LR [54], which lets the learning rate vary with epoch similar to the cosine function. The update strategy is demonstrated in Eq 11.

$$\eta_{t}=\eta_{min}+\frac{1}{2}(\eta_{max}-\eta_{min})\left(1+\cos\left(\frac{{Τ}_{cur}}{{Τ}_{max}}\pi \right)\right)$$ (11)

**Fig 6:**
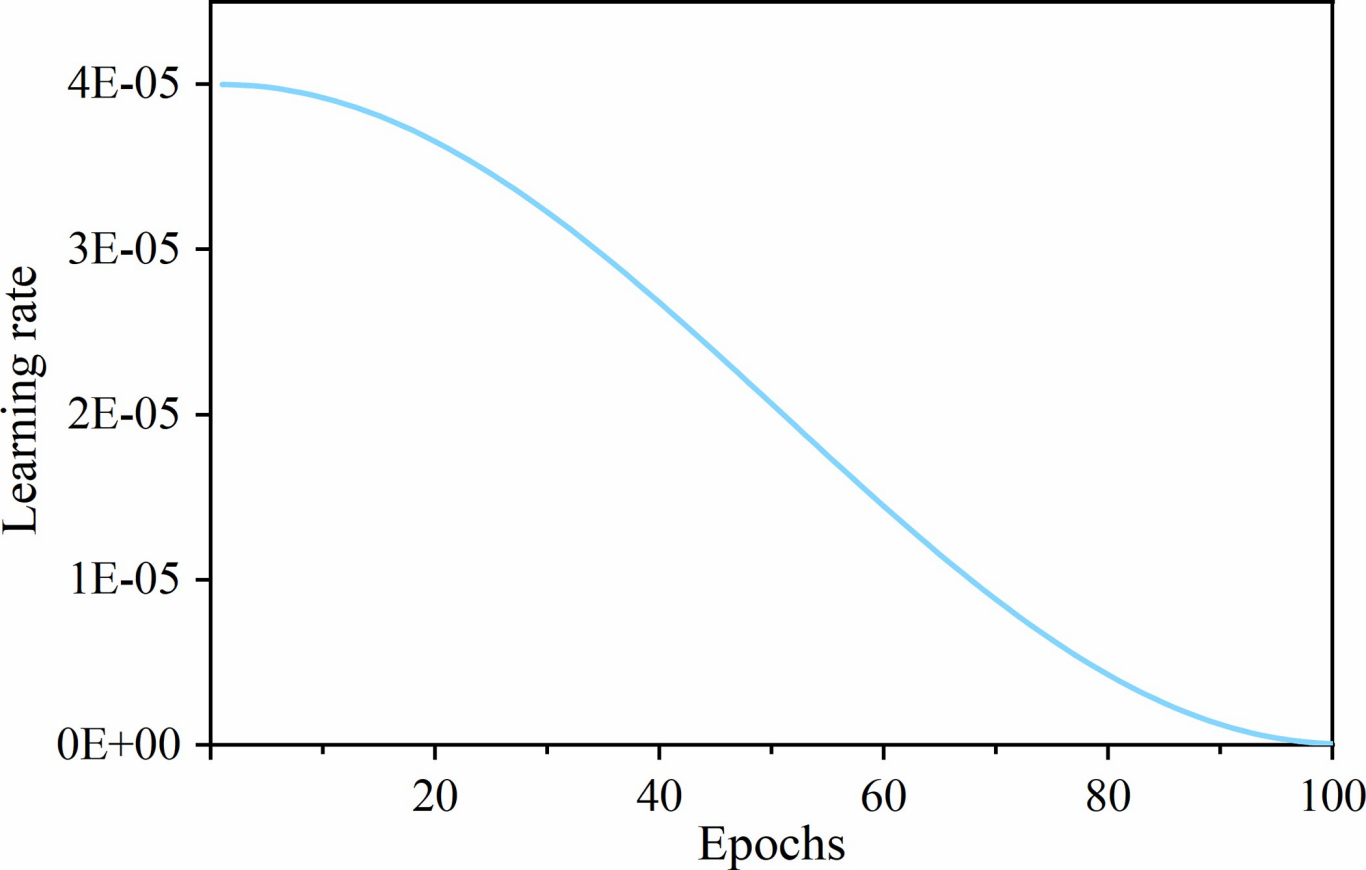
Diagram of learning rate decay. The maximum learning rate is 4e−5, the minimum learning rate is 1e−7, and the total number of epochs is 100.

Where, *η*_*max*_ is set to the initial learning rate (setting to 4e−5); *η*_*min*_ denotes the minimum learning rate (setting to 1e−7); *T*_*cur*_ is the current epoch; *T*_*max*_ is the maximum number of iterations, and the model has trained 100 epochs in this article. The learning rate strategy is shown in Fig 6.

#### 3.2.4 Transfer learning

**Fig 7:**
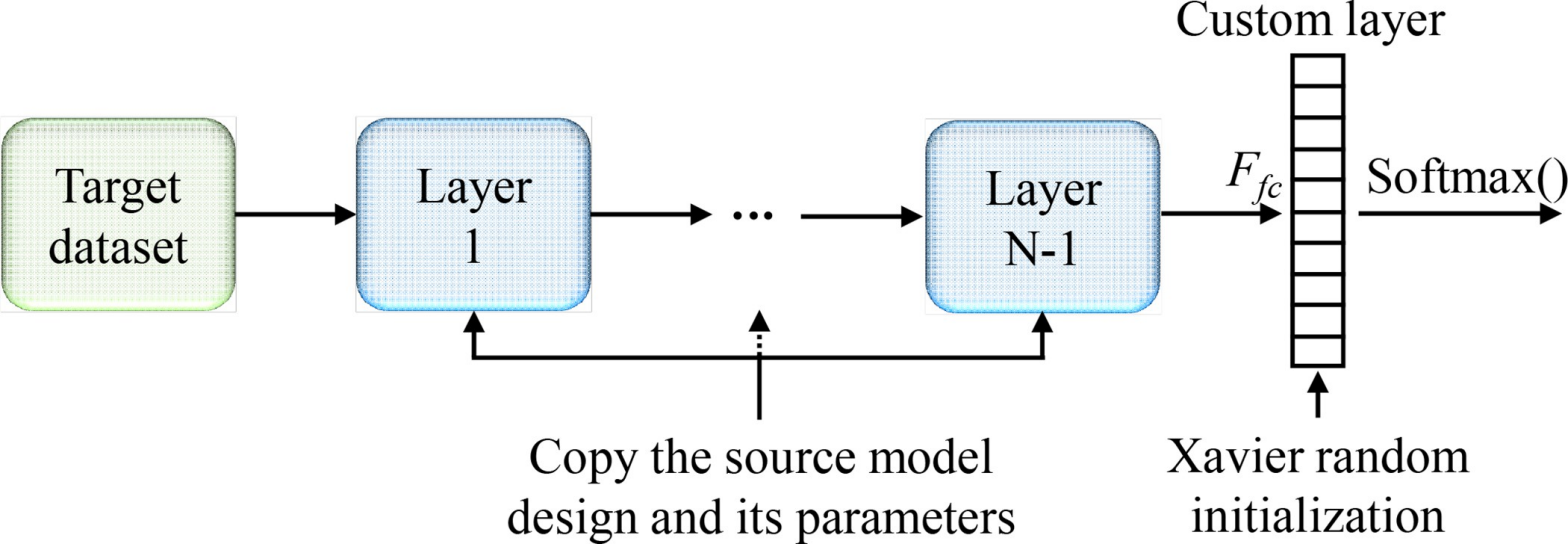
Fine-tuning. *F*_*fc*_ represents full connection. The weight parameters of the output layer were initialized by Xavier random initialization.

Transfer learning [55] can effectively improve the model accuracy while the training samples are limited. A common technique for transfer learning is fine-tuning, and Fig 7 shows its schematic. Except for the output layer, other network layers on the target model duplicate the source model network design and the corresponding parameters (the source model was pre-trained on ImageNet). In this work, the number of neurons in the output layer should be set to the number of lithology categories: 10, and its weight parameter was initialized by Xavier random initialization. The parameters of the output layer will be trained from scratch. To speed up the model convergence, a learning rate ten times that of the other layers was used in the output layer, and the parameters of other layers only need to be fine-tuned under the target task.

#### 3.2.5 Data augmentation

Plenty of training samples are the premise for training deep neural networks, and it is not easy to get drill core image data in underground engineering. Data augmentation creates similar more data by performing a series of random transformations on the training data, which increases the quantity of data and data diversity, and reduces the dependence of the network on some image attributes.

In this paper, upside-down or left-right turnover, random cropping, color change, etc. were implemented in the built-in online data augmentation method of the PyTorch framework, which preprocessed the loaded data with a certain probability during the training process, and does not change the amount of the original data. The drill cores are taken into pictures by the corresponding geological department. During the shooting process, different borehole cores are taken in different lighting environments, and the brightness, contrast, and saturation of the photos will be more or less different. Color is a very important feature for lithology identification, so the operation of randomly changing color in data augmentation is significant, which is more in line with the actual situation. In order not to distort the borehole image too much, we randomly changed the brightness, contrast, and saturation to 30% to 130% of the original image.

**Fig 8:**
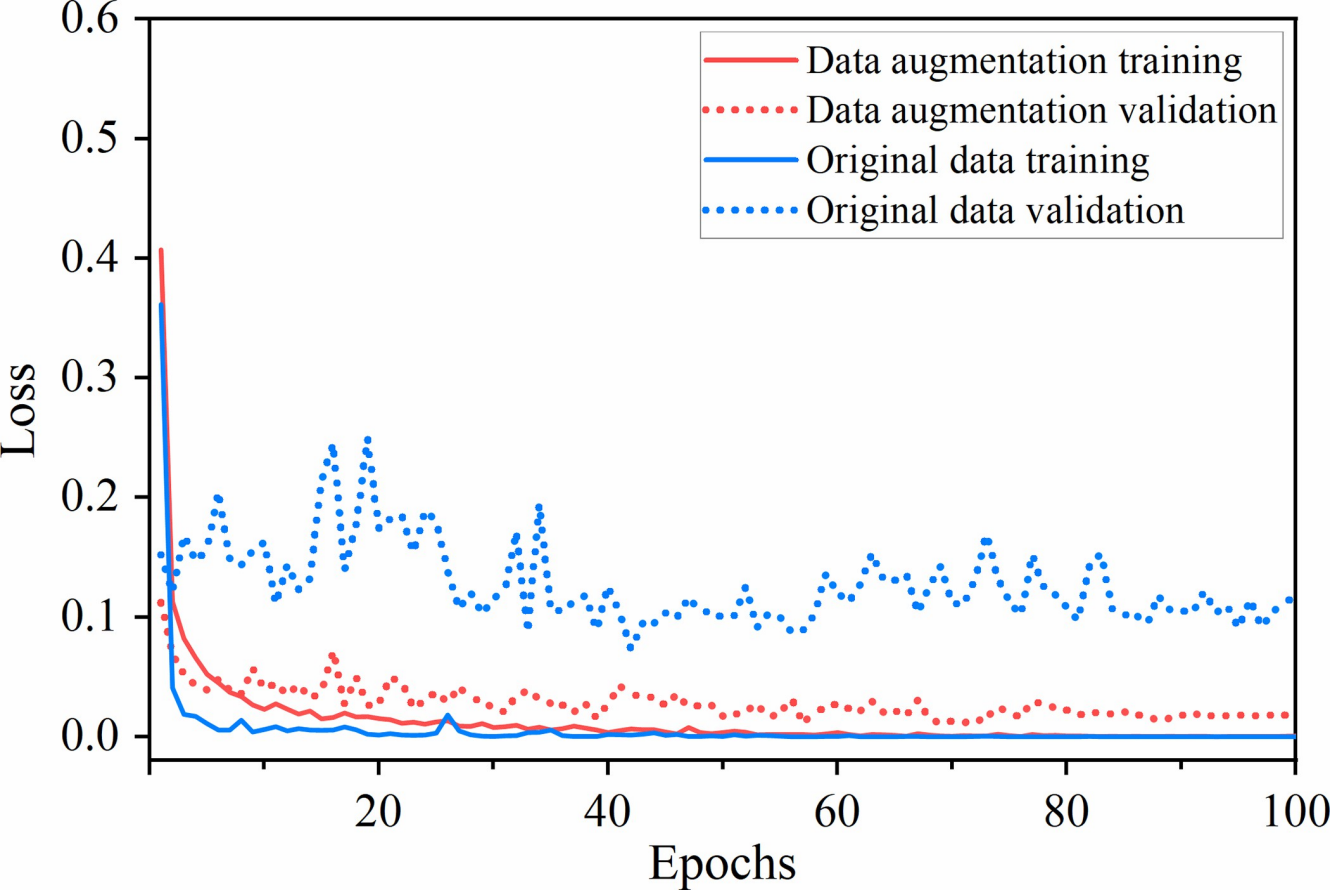
The effect of whether to use data augmentation on model training. Data augmentation can effectively alleviate overfitting and improve the generalization of the model.

The impact of the pre-trained ResNesSt-50 with or without data augmentation on model training was compared, as shown in Fig 8. During the training process of the original data, the training loss decreases quickly, but the validation loss decreases slowly, which is at a high level with more oscillations, and the model has serious overfitting as well. The figure suggests that the use of data augmentation for drill core images can effectively alleviate overfitting and improve the generalization ability of the model.

#### 3.2.6 Batch size

In the training process, the batch size is too small, the gradient oscillation is large, and the training time is long, besides, the performance of the device cannot be fully utilized. In contrast, the larger batch size reduces the noise in the gradient, and the model generalization is poor. In this paper, the mini-batches of 64, 32, and 16 images were set according to the GPU memory and were fed into the pre-trained ResNeSt-50 for experiments. The results are shown in Table 1. It is not a simple linear relationship between batch size and model accuracy. The accuracy of the model is higher for a batch size of 64 and decreases to varying degrees as the batch size decreases. Furthermore, the throughput of the CNN model decreases, and the total training time increases during this process. To fully utilize the device as well as maintain high prediction accuracy, the batch size was set to 64.

**Table 1:** Compare the experimental results of the model at three batch sizes.

Batch size	Test accuracy	img/sec	Total training time
64	0.9960	105.462	4 h 15m 7s
32	0.9947	94.828	4 h 40 m 54s
16	0.9957	76.783	5 h 23 m 53s

### 3.3 Evaluation metrics

For classification tasks, accuracy, precision, recall, and *F*_1−score_ are often selected as evaluation metrics to assess the performance of the architecture and show the superiority of the model. Accuracy indicates the proportion of correctly classified images to all images in testing group. Precision represents the proportion of correctly classified as positive images in all images judged by the classifier as positive. Recall denotes the ratio of correctly classified as positive images to all true positive images. *F*_1−score_ denotes the harmonic mean of precision and recall, which is used to measure the importance between precision and recall. These four metrics are given in Eqs (12)−(15), respectively.

$${Accuracy}_{v}=\frac{{TP}_{v}+{TN}_{v}}{{TP}_{v}+{FP}_{v}+{TN}_{v}+{FN}_{v}}$$ (12)

$${Precision}_{v}=\frac{{TP}_{v}}{{TP}_{v}+{FP}_{v}}$$ (13)

$${Recall}_{v}=\frac{{TP}_{v}}{{TP}_{v}+{FN}_{v}}$$ (14)

$${F_{1-score}}_{v}=\frac{2\times {TP}_{v}}{2\times {TP}_{v}+{FP}_{v}+{FN}_{v}}$$ (15)

Where, *v* is the lithology class label; TP is the number of images correctly classified as positive; TN is the number of images correctly classified as negative; FP is the number of images misclassified as positive; FN is the number of images misclassified as negative.

## 4 Experimental results

Three other well-known pre-trained models of VGG-13, ResNet-50, and DenseNet-161 in modern convolutional neural networks that are close to the performance of ResNeSt-50 were selected for training to achieve an apple to apple performance comparison with the pre-trained ResNeSt-50. According to the discussion about batch size in section 3.2.6, a mini-batch of 64 images in each epoch was fed to ResNeSt-50, ResNet-50, VGG-13 models, respectively. However, due to GPU memory constraints, DenseNet-161 received a mini-batch of 32 images. Data augmentation was performed on the images and then downsampling it to 224 × 224 pixels to match the input size of every architecture. All models used cross-entropy loss to guide model training. Besides, AdamW optimizer and Cosine Annealing LR strategy were used in all models to continuously update model parameters to reduce the loss value. The learning rate of ResNeSt-50 was set to 4e−5, and three other CNN models were slightly modified on the basis of 4e−5 according to their model characteristics to achieve the best fitting on the dataset.

### 4.1 Training, validation performance comparison

**Fig 9:**
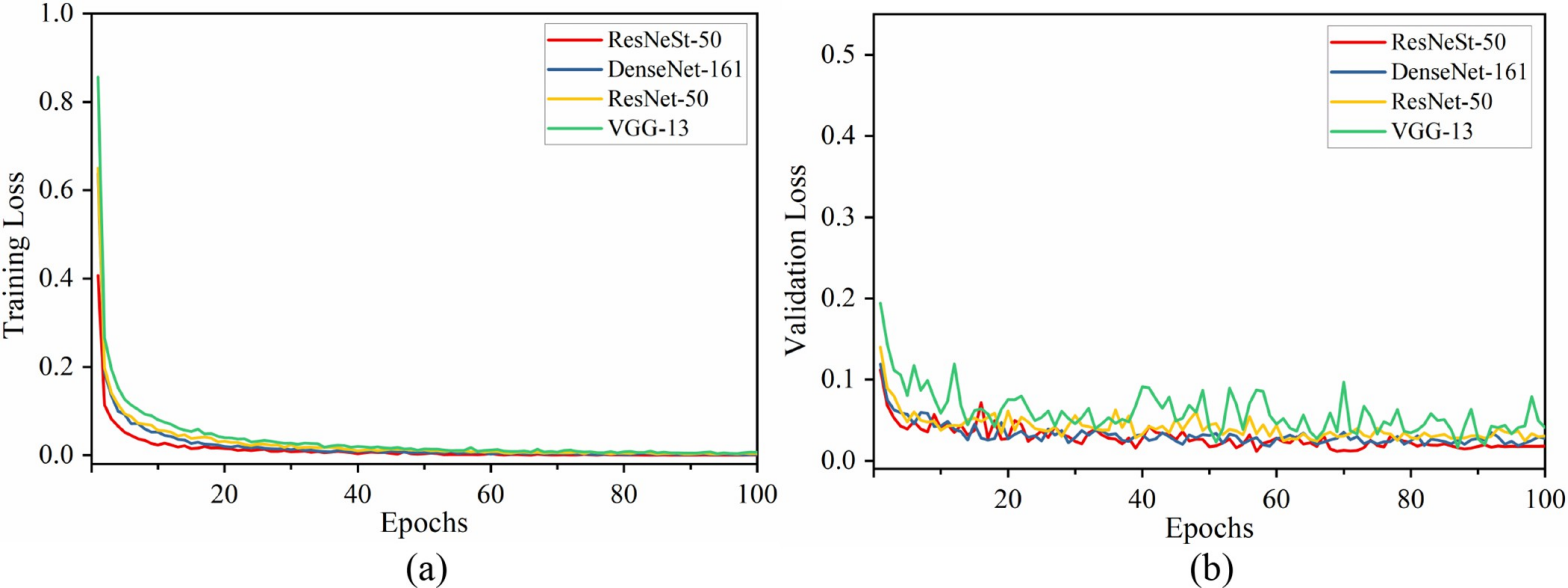
Total loss of four different CNN architectures during training (a) and validation (b).

**Fig 10:**
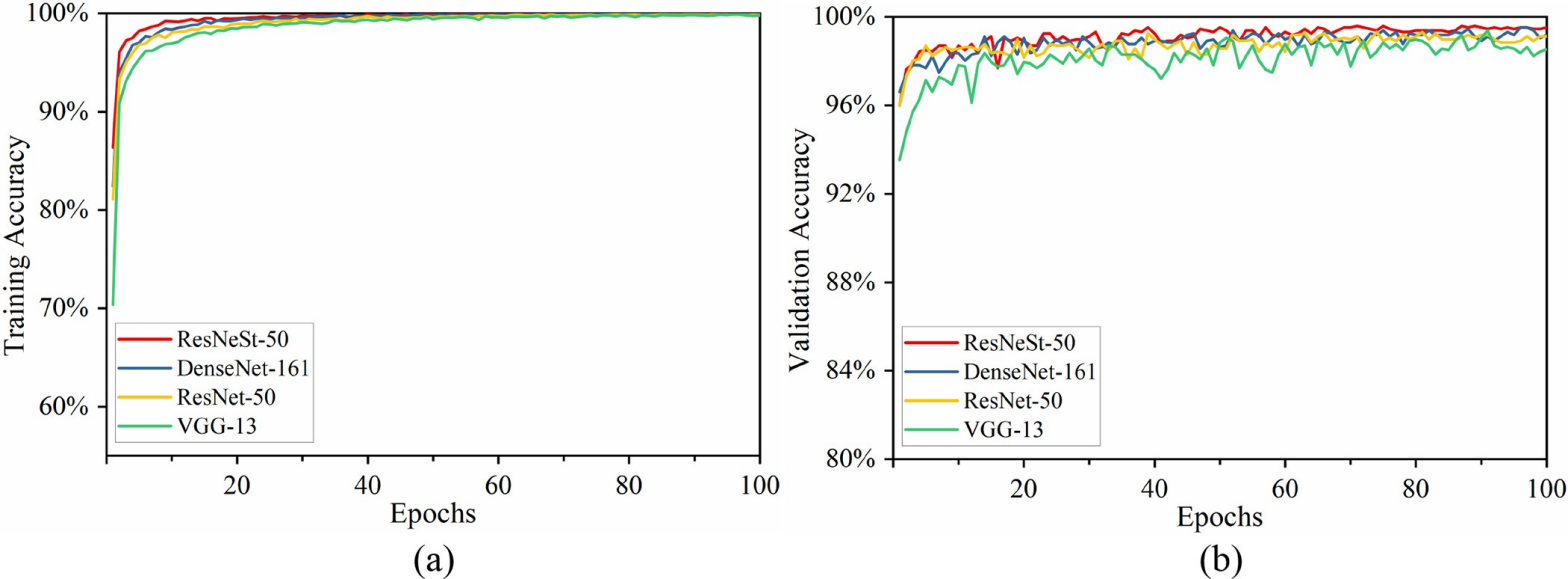
Accuracy of four different CNN architectures during training (a) and validation (b).

For this classification task, 10500 images were used for training, and 1500 images for validation. To evaluate the convergence and fitting effect of the model during training and validation, two indicators, total loss, and accuracy were used, and their changing process is shown in Figs 9 and 10, respectively. It shows that all four CNN architectures converge during training and validation. It can be seen from Fig 9(A) and 9(B) that the order of the total loss of the four CNN models, both on the training and validation process, from large to small is: VGG-13, ResNet-50, DenseNet-161, and ResNeSt-50. However, the four architectures exhibit the opposite of the total loss in terms of training accuracy and validation accuracy, in descending order: ResNeSt-50, DenseNet-161, ResNet-50, and VGG-13. In addition, in the stage of model convergence, the training loss of all four networks is smaller than their corresponding validation loss. While the training accuracy of all models is higher than their corresponding validation accuracy. ResNeSt-50 has the smallest total loss oscillation and accuracy oscillation throughout the training and validation process, indicating the strong robustness of the model.

It is worth noting that VGG-13 shows more obvious oscillations than the other three CNN models. The following two reasons might explain this phenomenon. For one thing, VGG-13 was pre-trained on ImageNet with 1000 categories, the three huge fully connected layers on the top layer have 4096, 4096, and 1000 neurons, respectively. while loading the pre-trained model, only the number of neurons in the output layer was changed to 10, and dropout isn’t used after each dense layer, which causes model overfitting, even if the L2 regularization term was added. For another, compared to three other architectures, VGG-13 doesn’t add a batch normalization layer inside the architecture, which makes it more sensitive to hyperparameters such as learning rate and batch size, and learning fluctuates greatly. Besides, without the batch normalization layer, to some extent, the regularization effect cannot be achieved. In conclusion, ResNeSt-50 is superior to three other typical models in this task.

### 4.2 Model evaluation on testing images

**Fig 11:**
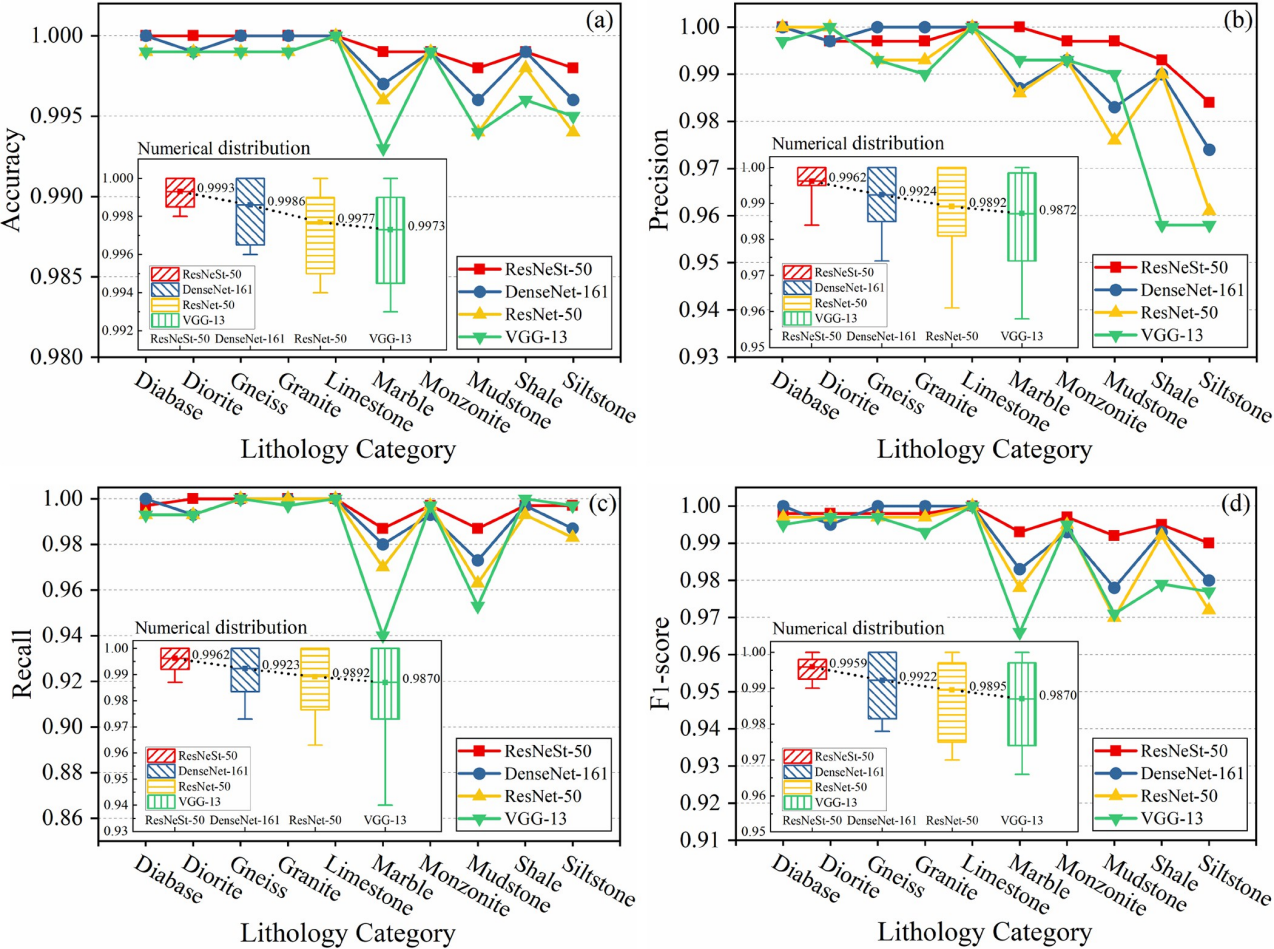
Performance of various lithology categories evaluated by (a) accuracy, (b) precision, (c) recall, and (d) *F*_1−score_, respectively. The subplots of each figure are the numerical distribution of each evaluation metric on each label, and the number next to the boxes represent the mean values.

According to section 2.4, 300 images (untrained images) were randomly chosen for each label and used for testing. The four metrics introduced in section 3.3 were used to compare the performance of the four CNN models on testing images, and the results are shown in Fig 11. It can be seen that the classification performance of the four CNN models on diabase, diorite, gneiss, granite, limestone, monzonite, and shale is roughly similar, and the performance of some other models is slightly better than ResNeSt-50 on individual categories, but ResNeSt-50 is larger overall in terms of the values of the four evaluation metrics. In addition, all CNN models have relatively poor classification performance on marble, mudstone, and siltstone, but the proposed method outperforms the three other methods on these three categories, and the four evaluation metrics values for these categories are significantly higher than those of the three other methods.

From the perspective of classifying all lithology categories, on the whole, in the four evaluation metrics, the position of the curve of ResNeSt-50 is above the other three CNN models. Our proposed model has a more concentrated numerical distribution for each lithology category on the Accuracy metric, with smaller variance and higher mean. The same rule is followed for the indicators precision, recall, and *F*_1−score_, with averages of 99.62%, 99.62%, and 99.59%, respectively. This further illustrates the excellent classification performance of ResNeSt-50, which can improve the classification accuracy of the drill core images dataset.

**Fig 12:**
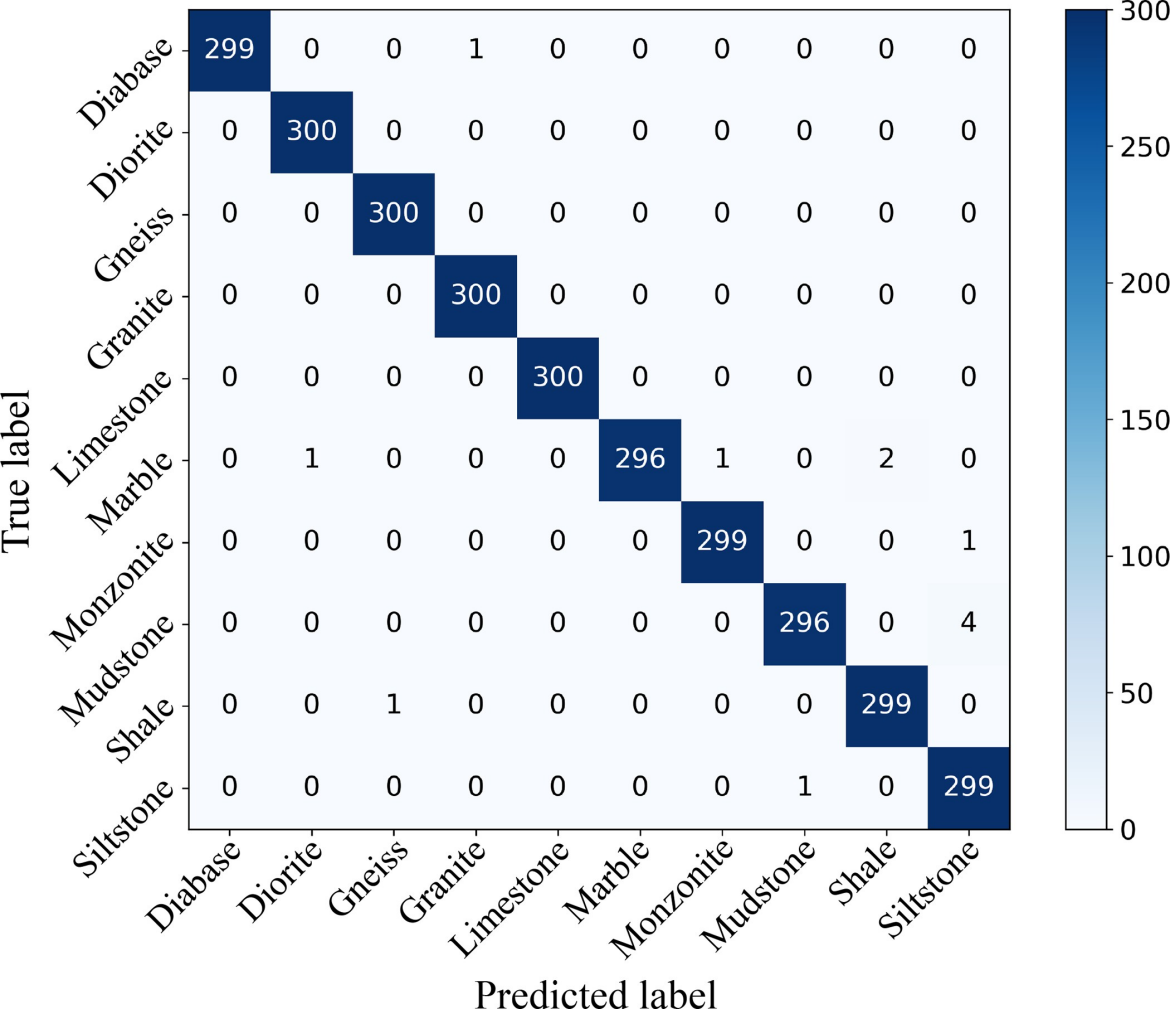
Confusion matrix of ResNeSt-50 on testing group.

**Fig 13:**
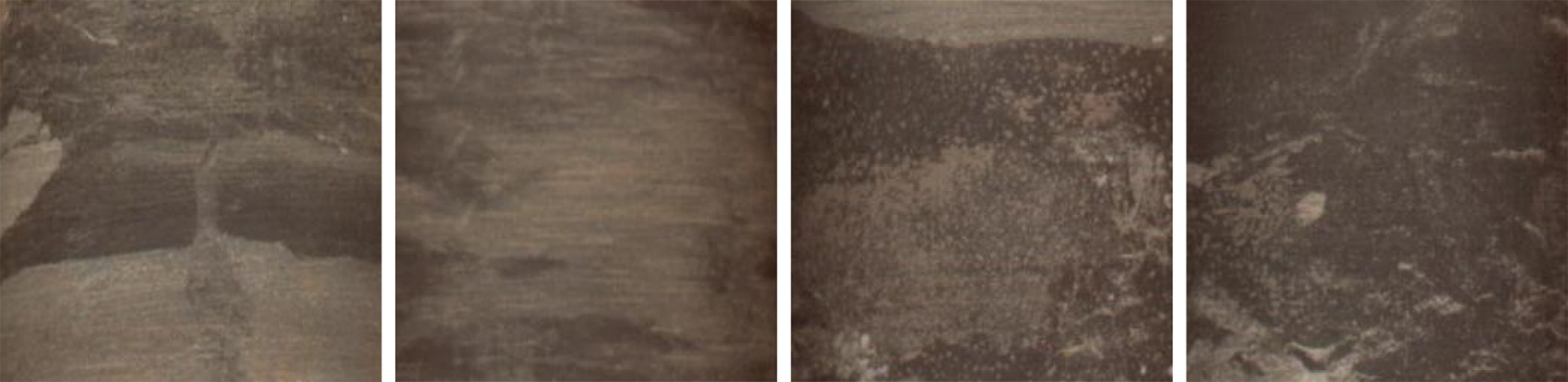
Images of mudstone misclassified as siltstone.

To further illustrate the prediction results of ResNeSt-50 on testing images, the confusion matrix shown in Fig 12 was used to visualize the results. In this article, it is a matrix of 10 rows and 10 columns, where each row represents the true class and each column represents the predicted class. The overall test accuracy is 99.60%, calculated as the sum of elements on the diagonal of the confusion matrix divided by the number of testing images. ResNeSt-50 performs the best classification for diorite, gneiss, granite, and limestone, and each category of the core images was correctly classified. The classification performance on diabase, monzonite, shale, and siltstone is second, with few images of each category of core images were misclassified. The network has the worst performance on marble and mudstone, which is consistent with the results discussed above. In the identification of marble, one image each was misclassified as diorite and monzonite. The reason is that the common mineral components of these three types of rocks are potassium feldspar, plagioclase, quartz, and calcite. Some parts of the drill core may appear similar color, structure, and texture, causing the model misjudgement. In addition, there are 2 images that were misclassified as shale. As can be seen from Fig 4, a few marble images have a similar color to shale, and a few core fractures of marble show the same layered structure as shale, which is likely to result in misjudgement. As for mudstone, 4 images were classified as siltstone, and their image examples are shown in Fig 13. It can be seen that mudstone and siltstone are very similar in color, rock structure and texture, and mineral compositions. In nature, two kinds of rocks are mixed with each other in the form of interbedded mudstone and siltstone, which often causes misidentification between them. Even experienced geologists have trouble distinguishing them with the naked eye, so the network misclassification is understandable.

### 4.3 Evaluation of other indicators

**Fig 14:**
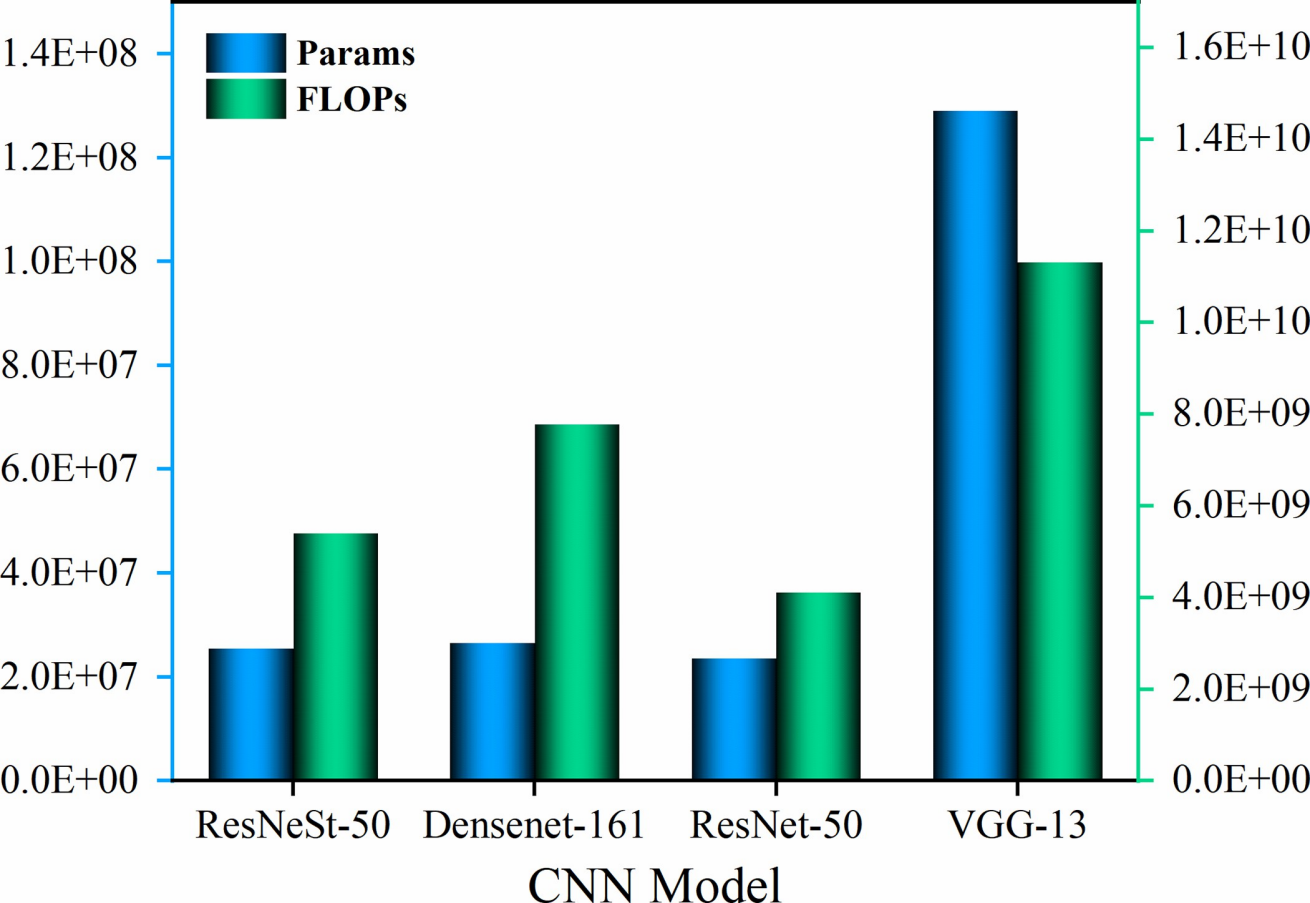
Comparison of Params, FLOPs of CNN models.

Params and FLOPs for the four CNN models are shown in Fig 14. It can be seen that the Params and FLOPs of all four models present the same trend, with the values following the same order from large to small: VGG-13, DenseNet-161, ResNeSt-50, and ResNet-50. ResNeSt-50 is generally at a lower level in terms of time and space complexity, only a small increase over the smallest numerical ResNet-50. In addition, several other indicators listed in Table 2 were also chosen to assess the model. As can be seen from the table, although ResNeSt-50 uses the strategy of combining channel-wise attention and multi-path network, its throughput during the training process still maintains a higher value, and the total training time only increases by about 1/3 compared with ResNet-50. During the testing, the inference speed (the average number of images processed per second with current GPU) of ResNeSt-50 ranks second. It is worth mentioning that ResNeSt-50 achieved the highest prediction accuracy 99.60% on testing images with only a small increase in the scale of parameters and model size compared to ResNet-50. Although DenseNet-161 achieved relatively high test accuracy, it took the longest time to train and had the lowest inference speed. Therefore, ResNeSt-50 achieves a better balance between model complexity and prediction accuracy, further demonstrating its applicability and excellent performance.

**Table 2:** Some other evaluation metrics were compared in the training process, testing process, and the model itself.

CNN Model	Training process		Testing process		Memory
	Throughput (img/sec)	Total training time	Inference speed (img/sec)	Test accuracy (%)	Model size (*M*)
ResNeSt-50	105.46	4 h 15 m 7 s	218.75	99.60	97.5
DenseNet-161	58.81	6 h 39 m 1 s	148.39	99.23	102.0
ResNet-50	170.42	3 h 8 m 6 s	265.89	98.93	90.0
VGG-13	124.97	3 h 53 m 24 s	201.59	98.70	492.0

In summary, ResNeSt-50 significantly outperformed DenseNet-161, ResNet-50, and VGG-13. The model had a lower total loss and higher accuracy during training and validation and provided superior classification performance in all lithology categories. In addition, the proposed network achieved very high prediction accuracy and provided high inference speed with a small increase in model complexity. Therefore, it can be concluded that ResNeSt-50 offers better performance on this drill core image classification task, and the model has better robustness and generalization performance.

### 4.4 Visual explanations of deep network

**Fig 15:**
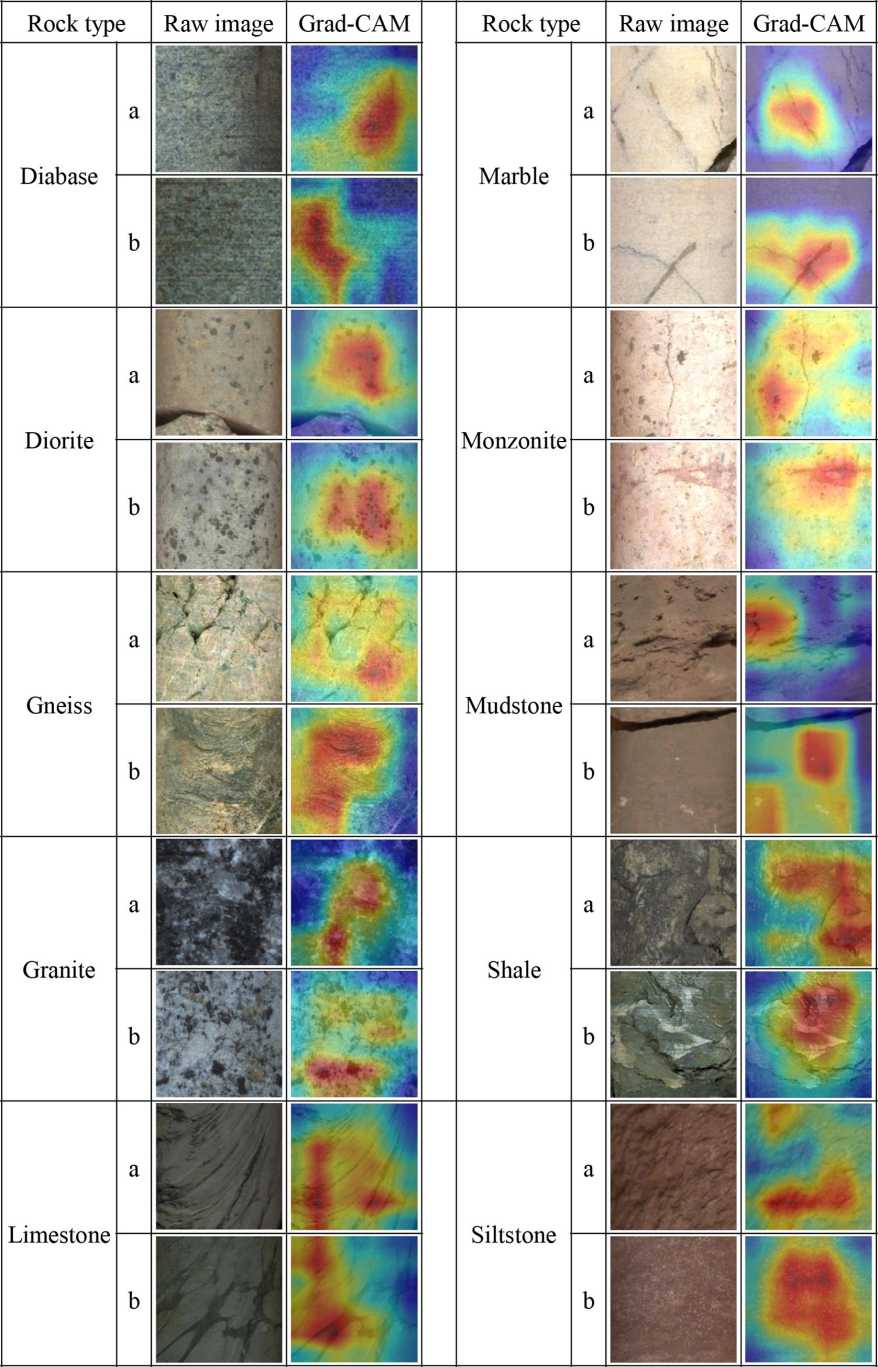
Grad-CAM visualization of DenseNet-161 making correct predictions on testing images.

CNN is like a black-box model. The convolutional layer as a feature extractor is mysterious in extracting features, and the addition of nonlinear activation functions makes it more non-transparent. Although CNN has achieved good classification results in lithology identification, its visualization and interpretability have always been a thorny problem. In this paper, we tried to use the Gradient-weighted Class Activation Mapping (Grad-CAM) [45] to visualize and explain the discriminative process of the model and explored the reasons why CNN predicts the input images as different categories of lithology. Its principle is to use the gradient information of the classification target (such as Diabase, Diorite, etc.) of the last convolutional layer of CNN architecture to generate a coarse localization, which is used to highlight the important regions of the image used to predict the target when the model is making decisions. Fig 15 shows Grad-CAM of typical image examples where the better interpretable DenseNet-161 made correct predictions. The highlighted regions (red regions) are the main focus areas of the network.

Rock color plays a crucial role in distinguishing lithology, but it can only roughly classify lithology, and rock structure, texture, and mineral composition are still the features that the model focuses on. The model accurately captured different rock structures, such as squamose or gneissic structures of Gneiss, layered structure in shale, and silty structure in siltstone. Different rocks exhibit distinctive rock textures, and the model can pay attention to these textures when making predictions, such as the gray-green texture in diabase, the unique texture in marble, and the oblique stripes in limestone. The mineral composition is the most essential property of rocks. The architecture can identify typical minerals in a discriminative way, such as chlorite in diabase, medium-fine-grained biotite in diorite, massive quartz and black mica minerals in granite, carbonaceous material in limestone, fine-grained dark minerals in monzonite, and white calcareous veinlet in mudstone. In addition, the model can also concern the developed cluster spots and dissolved pores in mudstone.

In general, visualization of CNN using Grad-CAM could reveal the key features that the model is more concerned with when making decisions, and Grad-CAM shows good interpretability in this classification task. However, the deeper semantic information of CNN models needs further exploration.

## 5 Discussion

The use of CNN for lithology identification has practical engineering application value. According to the experimental results, the following conclusions can be drawn: More advanced CNN model is essential for lithology prediction of drill cores, and the model tends to automatically extract more effective features under the same dataset. It is necessary to use advanced optimization algorithms because they can improve the efficiency of model training. Appropriate learning rate scheduling can reduce the model optimization divergence and make the model finally achieve better results. Data augmentation expands the original data scale and improves model generalization capability. The settings of hyperparameters such as learning rate and batch size are very important for training, and inappropriate training details will reduce the accuracy of the model.

Although the model we adopt has strong robustness and generalization performance, our work still has many shortcomings:

- Due to the lack of similar borehole data in China, our model has not been verified on new drill cores, and the robustness on similar datasets is unknown.
- The drill core images contain plenty of blurred pictures, red marks, and crushed structures, which are discarded in the experiment, resulting in images waste.
- Even with the same lithology, rocks still exhibit different characteristics in different stratigraphic environments. Therefore, the data scale of each type of lithology is relatively small, and the amount of data needs to be increased.

## 6 Conclusion

In this paper, a method based on fine-tuning techniques and convolutional neural networks was proposed to automatically predict the lithology of drill core images. ResNeSt-50 was used as the benchmark network, and three other representative CNN models were trained to compare the performance. The test results show that the ResNeSt-50, which uses a strategy of combining channel-wise attention with multi-path network, achieves a very good balance between model complexity and prediction accuracy. The network provided competitive performance in the lithology classification task of drill core images and achieved 99.60% prediction accuracy on untrained images. The following four main contributions were made in this research. Firstly, a dataset of drill core images of 10 common categories of lithology in underground engineering was established, and a classification scheme with engineering value and application prospects in the field of underground engineering was proposed. Secondly, a procedure was proposed to automatically process core images according to the input image size. Thirdly, the more advanced ResNeSt-50 was adopted as a feature extractor and classifier of drill core images. Lastly, an attempt was made to visually explain CNN in this task by Grad-CAM.

In the future, we will focus on the combination of training a machine learning algorithm classifier with well log data and training a CNN classifier with image data to predict core lithology using a voting mechanism and work on the construction of a digital platform for core analysis.

## Supporting information

S1 File (PDF)
