# Supplementary material for: Deep learning based lithology classification of drill core images
Source: PLoS One. 2022 Jul 1;17(7):e0270826. doi: 10.1371/journal.pone.0270826 (PMC9249224; doi:10.1371/journal.pone.0270826)
Supplement: S1 File — (PDF) [file pone.0270826.s001.pdf]

Supporting information

S1 Table. The individual data of accuracy in Fig 11(a).

Rock categories	CNN model			
	ResNeSt-50	DenseNet-161	ResNet-50	VGG-13
Diabase	1	1	0.999	0.999
Diorite	1	0.999	0.999	0.999
Gneiss	1	1	0.999	0.999
Granite	1	1	0.999	0.999
Limestone	1	1	1	1
Marble	0.999	0.997	0.996	0.993
Monzonite	0.999	0.999	0.999	0.999
Mudstone	0.998	0.996	0.994	0.994
Shale	0.999	0.999	0.998	0.996
Siltstone	0.998	0.996	0.994	0.995
Average	0.9993	0.9986	0.9977	0.9973

S2 Table. The individual data of precision in Fig 11(b).

Rock categories	CNN model			
	ResNeSt-50	DenseNet-161	ResNet-50	VGG-13
Diabase	1	1	1	0.997
Diorite	0.997	0.997	1	1
Gneiss	0.997	1	0.993	0.993
Granite	0.997	1	0.993	0.99
Limestone	1	1	1	1
Marble	1	0.987	0.986	0.993
Monzonite	0.997	0.993	0.993	0.993
Mudstone	0.997	0.983	0.976	0.99
Shale	0.993	0.99	0.99	0.958
Siltstone	0.984	0.974	0.961	0.958
Average	0.9962	0.9924	0.9892	0.9872

S3 Table. The individual data of recall in Fig 11(c).

Rock categories	CNN model			
	ResNeSt-50	DenseNet-161	ResNet-50	VGG-13
Diabase	0.997	1	0.993	0.993
Diorite	1	0.993	0.993	0.993
Gneiss	1	1	1	1
Granite	1	1	1	0.997
Limestone	1	1	1	1
Marble	0.987	0.98	0.97	0.94
Monzonite	0.997	0.993	0.997	0.997
Mudstone	0.987	0.973	0.963	0.953
Shale	0.997	0.997	0.993	1
Siltstone	0.997	0.987	0.983	0.997
Average	0.9962	0.9923	0.9892	0.987

S4 Table. The individual data of $F_{1-\text{score}}$ in Fig 11(d).

Rock categories	CNN model			
	ResNeSt-50	DenseNet-161	ResNet-50	VGG-13
Diabase	0.998	1	0.997	0.995
Diorite	0.998	0.995	0.997	0.997
Gneiss	0.998	1	0.997	0.997
Granite	0.998	1	0.997	0.993
Limestone	1	1	1	1
Marble	0.993	0.983	0.978	0.966
Monzonite	0.997	0.993	0.995	0.995
Mudstone	0.992	0.978	0.97	0.971
Shale	0.995	0.993	0.992	0.979
Siltstone	0.99	0.98	0.972	0.977
Average	0.9959	0.9922	0.9895	0.987
